# Hormonal and Metabolic Factors Influence the Action of Progesterone on the Endometrium of Women with Polycystic Ovary Syndrome

**DOI:** 10.3390/diagnostics13030382

**Published:** 2023-01-19

**Authors:** Maria Candida P. Baracat, Edmund C. Baracat, Ricardo S. Simões, Manuel J. Simões, Gustavo A. R. Maciel, Ricardo Azziz, José Maria Soares

**Affiliations:** 1 Disciplina de Ginecologia, Departamento de Obstetrícia e Ginecologia, Hospital das Clínicas HCFMUSP, Faculdade de Medicina, Universidade de São Paulo, Sao Paulo 05403 000, Brazil; 2 Department of Obstetrics & Gynecology, School of Medicine, University of Alabama at Birmingham, Birmingham, AL 35294, USA; 3 Department of Health Policy, Management, and Behavior, School of Public Health, University at Albany, SUNY, Albany, NY 12222, USA

**Keywords:** polycystic ovary syndrome, endometrium and progesterone treatment

## Abstract

Hormonal and metabolic factors may influence endometrial quality and interfere with the action of progesterone. Therefore, the aim of our study was to address this issue. Participants were recruited from an outpatient reproductive endocrinology clinic at an academic tertiary medical care centre. All subjects underwent endometrial biopsy (EB) in the follicular phase of the cycle prior to treatment. Thereafter, they were treated with micronized progesterone (400 mg/day × 10 days intravaginally) from days 14–28 of the next cycle. A second EB was performed between days 21–24 of the cycle (the second phase). The metabolic and hormonal serum levels were evaluated during the implantation window. EB samples were analysed using light microscopy for histomorphometric analysis. The endometrium of women with Polycystic Ovarian Syndrome (PCOS) in the second phase demonstrated a uniform surface epithelium with less leukocyte infiltration and an absence of apoptotic figures compared to the control group. (*p* < 0.021). The thickness of the surface epithelium in the second phase of the PCOS group correlated positively with free and bioavailable testosterone values. The number of stromal cells increases with increasing insulin levels. Our results suggest that histomorphometric abnormalities of the endometrium persist and are linked to androgen and insulin levels despite progesterone supplementation in PCOS.

## 1. Introduction

Polycystic ovary syndrome (PCOS) is a complex endocrine and reproductive condition that is associated with chronic anovulation and/or hyperandrogenism and polycystic ovaries. The diagnosis is made by the absence of other pituitary or adrenal disorders, such as hyperprolactinemia, hypothyroidism, acromegaly, Cushing’s syndrome, and enzyme deficiency of the adrenal gland in its non-classical or late form. It can be presumed that the frequency of the syndrome varies greatly, from 5% to 20% [1,2,3,4,5].

Given the state of chronic anovulation, the syndrome poses two great endometrium-linked dilemmas: the risks of infertility and proliferative lesions [6,7,8]. Progesterone action on the endometrium is related to androgen receptor regulation in the epithelium and endometrial stroma [6]. Progesterone acts principally 7–10 days after ovulation, when the endometrium is more receptive to embryo implantation during the implantation window. Such a process is critical for mother-embryo interactions [4,9,10,11,12]. However, high androgen levels may be harmful to progesterone action, decidualization, and endometrial receptivity [3,4]. Furthermore, a PCOS-related chronic inflammatory process, along with carbohydrate metabolism disorders, is viewed as a factor that hampers mother-embryo interaction and trophoblast invasion, possibly leading to miscarriage [3,4,11,12]. Nevertheless, there is a need to confirm the role of hyperandrogenism in progesterone action and its association with metabolic disturbances such as lipids and insulin resistance [11]. In addition, metabolic complications in PCOS patients, such as insulin resistance, may be related to a local oxidative stress imbalance in the endometrium, leading to poor endometrial receptivity and effects on pregnancy [6]. Chronic inflammation in PCOS, involving interleukin (IL)-6, IL-16, IL-18, tumour necrosis factor (TNF) -α, and C-reactive protein (CRP), may play a role in the endometrial oxidative stress imbalance as well as the upregulated expression of intercellular adhesion molecule 1, TNF-α, and metalloproteases. Furthermore, obesity combined with metabolic disorders and gut microbiota imbalance can lead to endometrial OS imbalance through downregulation of the oestrogen receptor (ER), which is important for the expression and activity of the progesterone receptor (PR). Therefore, it may affect the endometrial receptivity process [12].

Simões et al. [13] observed that the endometrium of women with PCOS had a different protein composition, such as hyaluronic acid, compared to the endometrium of women without PCOS. In another study, Simões et al. [14] found alterations in the composition of low molecular weight proteoglycans (SLRP) in the extracellular matrix of the endometrium of women with PCOS, which could predispose to the development of endometrial proliferative lesions such as hyperplasia and cancer.

Literature shows that ovulation induction in women with PCOS does not necessarily lead to improved endometrial receptivity and trophoblast invasion in assisted reproductive technology programs. For instance, treatment with clomiphene citrate results in relatively low pregnancy rates in PCOS, despite ovulation rates of up to 82% [15,16,17]. This fact has also been confirmed in women with PCOS who underwent in vitro fertilisation, whose success rates were also lower (25–45%) than those in non-PCOS patients [17,18]. These data suggest that anovulation is not the only cause of infertility in PCOS [3,4,7,8,9,15,19,20,21,22,23]. 

These findings suggest that there might still be other factors influencing endometrial quality or interfering with progesterone action. We hypothesised that the conventional dose of progesterone (400 mg/day) administered by the vaginal route may not be enough to correct all histological aspects of the endometrium related to PCOS. Second, the metabolic and hormonal profiles of PCOS patients may interfere with progesterone’s action on the endometrium. Therefore, this study aimed to evaluate the effect of micronized progesterone administered through the vaginal route on the endometrium and the possible interference of these factors on the tissue response to progesterone.

## 2. Materials and Methods

### 2.1. Subjects

The inclusion criteria for PCOS women were as follows: (1) aged 18 to 35 years, and (2) PCOS was defined by the Rotterdam criteria, which includes two of the following three features: (a) oligo-amenorrhoea, (b) clinical or biochemical signs of hyperandrogenism, and (c) polycystic ovaries [3]. These criteria yielded four PCOS phenotypes (A–D) [1,2,5]. Controls were recruited from a family planning outpatient office. All participants were healthy women aged 18–35 years with regular menstrual cycles, no evidence of hirsutism, no comorbidities, and no use of hormone medications in the previous 3 months. 

Women with other causes of hyperandrogenism or anovulation were excluded using appropriate tests, and participants had not used hormonal or other drugs in the three months prior to the study. Women with systemic diseases, sexually transmitted diseases, uterine tumours, ovarian cysts or tumours, other endocrine disorders in addition to PCOS, or use of statins, corticosteroids, or infertility drugs in the 3 months prior to the study were excluded. Women positive for the β-subunit of human chorionic gonadotropin (β-HCG) were also excluded.

**Figure 1:**
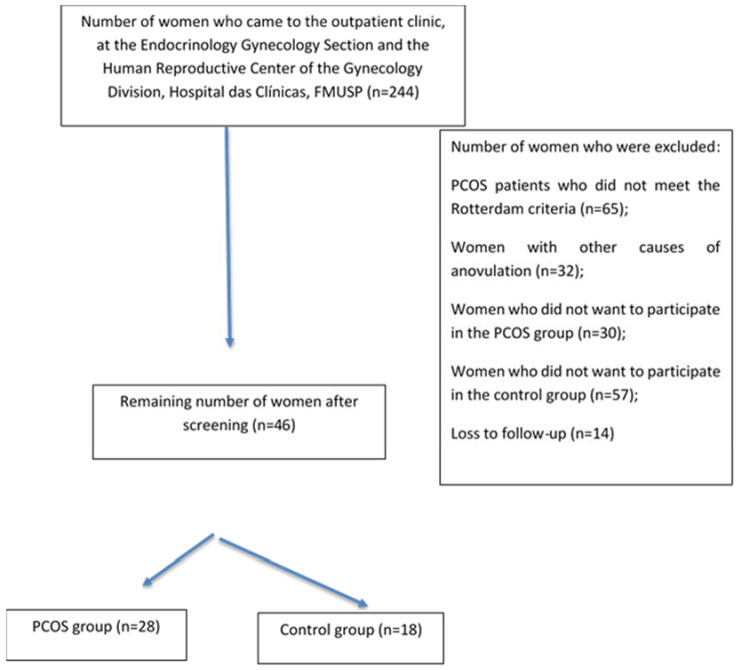
Flow chart of the study.

The flowchart of the study is shown in Figure 1. Patients were evaluated at the Gynecology Outpatient Office. After applying the eligibility criteria and explanation of the protocol to 244 women who were recruited by electronic media or came from the family planning outpatient office, 46 were selected and divided into two groups: (a) women with PCOS (n = 28) and (b) controls (n = 18). All patients were cared for at the Endocrinology and Gynecology Section and Human Reproductive Center of the Gynecology Division, Hospital das Clinicas, FMUSP (Figure 1). The reasons for exclusion were as follows: patients with PCOS who did not meet the Rotterdam criteria (n = 65), women with outer causes of anovulation (n = 32), women who did not want to participate in the PCOS group (n = 30), women who did not want to participate in the control group (n = 57), and loss to follow-up (n = 14). 

### 2.2. Study Protocol

This prospective open-label trial was approved by the Research Ethics Committee (#21253613.6.0000.0068) of the Faculdade de Medicina Hospital das Clinicas HCFMUSP, Universidade de São Paulo (FMUSP), São Paulo, Brazil. Participants were recruited from an outpatient reproductive endocrinology clinic at an academic tertiary medical care centre (Human Reproduction Center, Gynecology Division, Hospital das Clinicas, FMUSP) between 2014 and 2020 (clinical trial number: NCT05062135). The study was conducted in accordance with the Declaration of Helsinki for medical research involving human subjects. After signing an informed consent form written in accordance with Resolution 196/96 of the National Health Board (Resolução 196/96 do Conselho Nacional de Saúde), all subjects were included in the study and evaluated as follows.

### 2.3. Preselection

This visit included the following: interview for medical history; overall physical and gynecologic examinations; weight measurement (scale Filizolla, model PL-150, with a capacity of 150 kg); height measurement with a metal stadiometer attached to the scale; abdominal circumference (AC) measurement with a measuring tape at the narrowest region between the last costal arch and the iliac crest; hip circumference (HC) measured around the circumference of the hips at the top of the iliac crest and buttocks; body mass index (BMI); blood pressure (BP) measurement; and hirsutism according to the modified Ferriman-Gallwey (mFG) scoring system with nine areas, with patients with a score >8 deemed as hirsute. 

The menstrual cycle was considered normal when the interval between the periods was 21 to 35 days and the blood flow lasted no more than 7 days. The cycle was characterised as anovulatory when the span between blood flows was >35 days [1,2,3,4,5]. Amenorrhoea was defined as the absence of menstrual flow for a period corresponding to three previous cycles or for >90 days [1,2,3,4,5].

Patients were asked to fast for 12 h prior to their visit to the Central Laboratory of Hospital das Clínicas. Blood was collected, and transvaginal pelvic ultrasound was performed on all study subjects. 

We realized two studies: PCOS-Study A and PCOS-Study B. 

#### 2.3.1. PCOS-Study A

**Figure 2:**
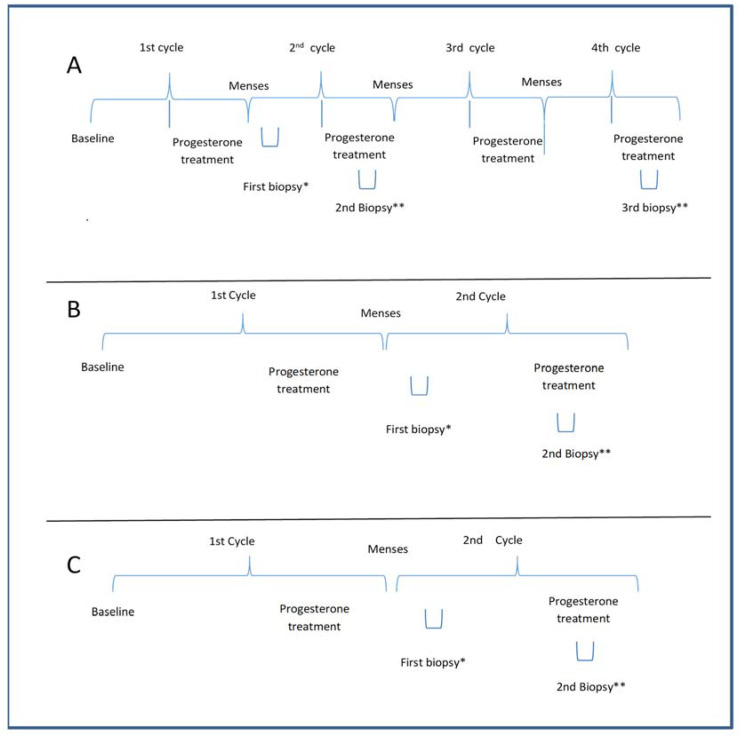
(**A**) The protocol for evaluating the effect of the number of cycles of progesterone on the endometrium in PCOS in Study A. The second and third biopsies were performed under progesterone treatment in the pilot study; (**B**) The protocol of treatment and biopsy for PCOS in Study B; (**C**) The protocol of treatment and biopsy for the control group in Study C. Baseline of A and B: Patients having amenorrhea with negative beta HCG; Baseline of C: patients with regular menses without any PCOS features; a progesterone treatment period with 400 mg micronized progesterone through the vaginal route; * the first biopsy was performed during the 5th to 9th day of the menstrual cycle; ** the biopsy was done during the progesterone treatment during the 20th to 24th day of the menstrual cycle.

The PCOS-Study A was designed to assess whether progesterone could transform proliferative into secretory endometrium, regardless of the number of treatment cycles. Therefore, six PCOS patients with amenorrhoea (>90 days) and negative β-HCG were prescribed micronized progesterone (400 mg) administered daily via the vaginal route for 10 days. Subsequently, under the second treatment cycle with natural progesterone (400 mg, vaginally, for 10 days), two endometrial biopsies were performed between days 5 and 9 and between days 21 and 24 of the menstrual cycle (day 1 being the first day of vaginal bleeding). In the next cycle, after progesterone treatment, a third biopsy was performed between days 21 and 24 (Figure 2). 

#### 2.3.2. PCOS-Study B

Twenty-two PCOS subjects underwent two endometrial biopsies after the first progesterone menstrual cycle, the first between days 5 and 9 of the cycle and the other between days 21–24 (i.e., under progesterone treatment) of the cycle (Figure 2). This study assessed whether endometrial dysfunction persists after progesterone supplementation.

Controls: Eighteen women were used as controls and underwent two endometrial biopsies after the first progesterone treatment, the first between days 5 and 9 and the second between days 21–24 of the cycle (Figure 2).

### 2.4. Assays

The oral glucose tolerance test (OGTT) consisted of 75 g of glucose administered orally after three days of a carbohydrate-rich diet; blood samples for glucose and insulin measurements were drawn before administration of the glucose solution and 30, 60, 90, and 120 min after administration.

Plasma glucose concentration was determined using the glucose oxidase method [24]. All lipid measurements were obtained directly from plasma samples. The total cholesterol and triglyceride levels were assessed using enzymatic methods (Roche Laboratories). HDL-C was quantified using the same method, and LDL-C was estimated using the Friedwald formula [25]. Glycated hemoglobin was measured using high-performance liquid chromatography [25] in a centrifuge. Progesterone was measured using an immunofluorometric assay (Wallac, Helsinki, Finland) using Auto DELFIA kits; androstenedione, prolactin (PRL), luteinizing hormone (LH), and follicular stimulating hormone (FSH), by the immunofluorometric assay; dehydroepiandrosterone sulfate (DHEAS), by radioimmunoassay (Cisbio International, Saclay, France, and DSL, Houston, TX, USA); insulin and 17-OHP, by radioimmunoassay using DSL kits. Testosterone and sex hormone-binding globulin (SHBG) levels were measured using an electrochemiluminescent immunoassay (Modular; Roche). The free testosterone index was calculated using the following formula: total testosterone/SHBG × 100. Free testosterone levels were calculated using the Vermeulen formula. All analyses were performed twice, and the intra- and inter-assay coefficients of variation did not exceed 10% and 15%, respectively. 

### 2.5. Endometrial Biopsies

Endometrial samples were obtained using a Pipelle™ de Cornier catheter (Laboratoire CCD; Paris, France). The tissue was embedded in paraffin for histomorphometric analysis. 

Endometrial samples were fixed for 24 h in 10% formaldehyde, dehydrated in increasing concentrations of ethyl alcohol, cleared in xylene, and embedded in paraffin. Paraffin-embedded samples were cut into ten 3-μm sections per subject. Four sections were stained with hematoxylin and eosin to confirm the menstrual cycle phase (proliferative) using the criteria suggested by Noyes and Haman [26] and morphometry.

### 2.6. Morphological and Histomorphometric Analyses

To quantify the parameters of interest, images were captured using a high-resolution camera (AxioCam-MCR, Carl Zeiss, Jena, Germany) adapted to a light microscope (Axiolab, Carl Zeiss) and adjusted using 40× objective lenses. The images were transmitted to a computer using AxioVision Rel 4.2 software (Carl Zeiss). For the assessment of glandular and surface epithelial thickness, cell count, and blood vessels, ten images of each endometrial sample were obtained for each patient. 

### 2.7. Statistical Analyses

The G power program (https://www.statisticssolutions.com/free-resources/sample-size-power-analysis accessed on 2 December 2021) was used for power calculation in Study B. Histomorphometric data from Study A were used to calculate the sample size. The thickness of the surface epithelium is widely used for this purpose. For this, a significance level (alpha) of 0.05 and a power of 80% were considered, based on the results of a pilot study (Study A), whose mean was 21.33 µm in the PCOS group and 15.91 µm in the control group, with a standard deviation of 12.88 µm and an effect size of 0.51, resulting in a total of 30 participants (15 per group). 

Commensurate with homogeneity, quantitative data are expressed as the mean ± standard deviation. The PCOS patients in Study A were grouped with those in Study B for the final analyses. In conformity with the data distribution, the Student’s *t*-test or Mann–Whitney *U* test was used. Moreover, analysis of variance, or the Kruskal–Wallis test, was used for histologic and immunohistochemical comparisons when an additional group (PCOS during the proliferative phase) was included. Thereafter, in line with the data distribution, either Tukey’s test or Dunn’s test was applied. Spearman’s correlation was applied for continuous clinical and laboratory variables with histological data. The significance level was set to 95% (α ≤ 5%).

## 3. Results

### 3.1. Clinical Data

Of the 46 women included, 28 (60.87%) had PCOS, and 18 (39.13%) were controls. Table 1 displays a summary of the clinical and sociodemographic data of the study participants. The mean ages of the participants in the PCOS and control groups were 27.41 ± 5.73 years and 30.11 ± 5.31 years (*p* = 0.121), respectively. No statistical differences were found between the groups in terms of menarche, ethnicity, marital status, schooling, family income, religion, duration of menstrual flow, and number of childbirths. However, there was a significant difference in the number of yearly menstruations and pregnancies. Women with PCOS had a lower number of menstrual cycles yearly (3.18 ± 2.23) and a lower number of pregnancies (0.19 ± 0.41) compared to those in controls (11.63 ± 0.42, *p* < 0.001; and 0.72 ± 0.55, *p* = 0.022, respectively). 

### 3.2. Metabolic Data

In Table 2, anthropometric data as well as data on systemic blood pressure and glucose and lipid metabolism are depicted. There were no significant differences between the groups in terms of BMI or AC. The hip circumference was higher in women with PCOS than in controls (*p* < 0.01). 

**Figure 3:**
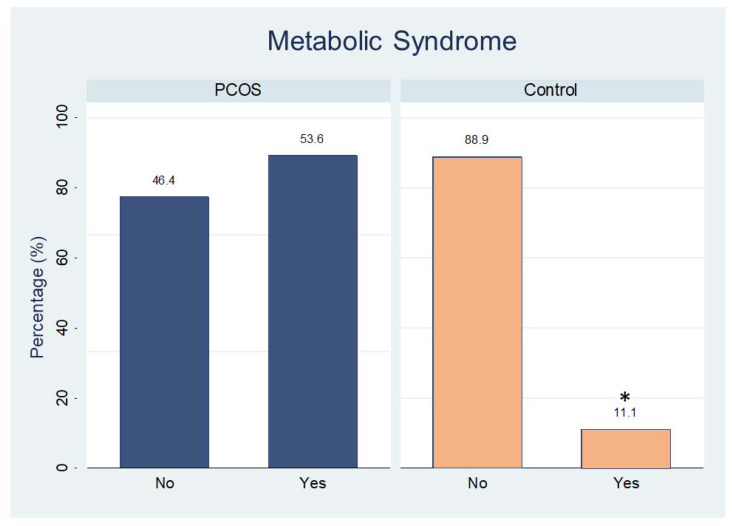
Percentage of participants with (yes) or without (no) metabolic syndrome in both groups. * *p* < 0.01.

Analysis of carbohydrate metabolism demonstrated that women with PCOS had higher values than controls for the following: glycated hemoglobin; OGTT (glucose measurements at 30 min, and 60 min); and insulin tolerance test (insulin at 0 min (fasting), 60 min, and 120 min); HOMAIR and Matsuda index. The other carbohydrate values of lipid metabolism did not differ significantly between the groups, except by HDL-cholesterol and triglycerides levels (Table 2). The percentage of women with metabolic syndrome in the PCOS group was higher than that in the control group (Figure 3, *p* = 0.01).

### 3.3. Ferriman–Gallwey Index and Laboratory Data

Table 3 presents the clinical data on hirsutism based on the mFG score as well as the complementary hormone measurements and the free and bioavailable testosterone indices. The mFG score, total testosterone, free testosterone fraction, percentage of free testosterone fraction, bioavailable testosterone (ng/dL), percentage of bioavailable testosterone, and androstenedione were significantly higher in the PCOS group than in the controls. However, the opposite was true in relation to SHBG; the PCOS group had a significantly lower level (30.3 ± 14.7 nmol/L) than the control group (71.61 ± 45.11 nmol/L, *p* < 0.001).

The levels of the gonadotropic hormones FSH and LH were higher and lower, respectively, in women with PCOS than in controls (*p* < 0.01). No other significant differences were found between the groups with respect to DHEAS (dehydroepiandrosterone sulfate), oestradiol, and PRL levels (Table 3).

### 3.4. Histomorphologic Analysis

**Figure 4:**
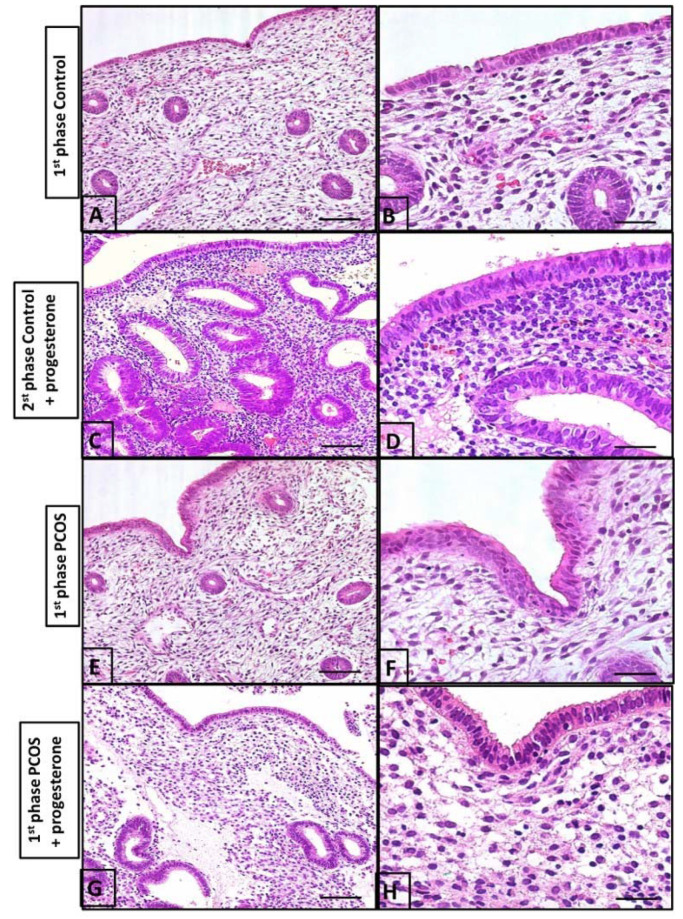
Histological evaluation of the endometrium. (**A**) the surface shows simple columnar epithelium and the endometrial coiled glands during the first phase of the menstrual cycle; (**B**) the lamina propria is rich in blood vessels and intercellular substances during the first phase of the menstrual cycle; (**C**) the second phase of the menstrual cycle upon undergoing progesterone treatment showing numerous endometrial glands compared to those in (**A**) (1st phase of cycle), with a high number of cells in the stroma; (**D**) the second phase of the menstrual cycle upon undergoing progesterone treatment columnar epithelium; (**E**) the endometrium of a PCOS patient. Note the cell-rich lamina propria is slightly more developed than in (**A**,**B**); (**F**) the endometrial gland and vessels are similar to (**A**,**B**); (**G**) leukocytes in the stroma are numerous, with lesser endometrial glands than in the control group (**C**,**D**); (**H**) few endometrial glands and pseudo-stratified epithelium. These findings are different from (**C**,**D**). Bars in (**A**,**C**,**E**,**G**) and in (**B**,**D**,**F**,**H**), are 40 µm and 5 µm, respectively.

The control group presented normal histological features for the first phase (proliferative) of the menstrual cycle as well as the second phase (secretory) (Figure 4). PCOS-Study A did not indicate significant histological changes when comparing the second and fourth progesterone treatment cycles. Therefore, we decided to perform a biopsy during the second cycle of the main protocol.

The control biopsy performed between the 5th and 9th days of the cycle demonstrated an endometrium covered with simple columnar epithelium containing small elevations at the apical pole and countless coiled tubular glands in the lamina propria. It should also be noted that the lamina propria contained sparse cells with numerous blood vessels (Figure 4A,B). The second biopsy performed between the 21st and 24th day of the cycle in controls who had received progesterone demonstrated a high number of endometrial glands and a high number of cells in the stroma (Figure 4C,D).

In women with PCOS, biopsy between the 5th and 9th days of the cycle following progesterone treatment in the prior cycle demonstrated an endometrium with essentially the same characteristics as those of controls during the proliferative phase of the menstrual cycle. However, the surface and glandular epithelia were thicker, and the lamina propria, situated beneath the surface epithelium, was rich in cells and leukocytes. Intense leukocyte infiltration in the surface epithelium as well as a few apoptotic figures were observed when compared to the control group (Figure 4). The endometrium of women with PCOS receiving progesterone (between the 21st and 24th day of the cycle) demonstrated an overall more uniform surface epithelium with less leukocyte infiltration and an absence of apoptotic figures. Numerous branched tubular glands were observed in the lamina propria, along with a large concentration of cells. Overall, the endometrium during days 5 and 9 of the cycle in PCOS patients (Figure 4) was slightly more developed than that in the control group (Figure 4A,B).

### 3.5. Histomorphometric Analysis

**Figure 5:**
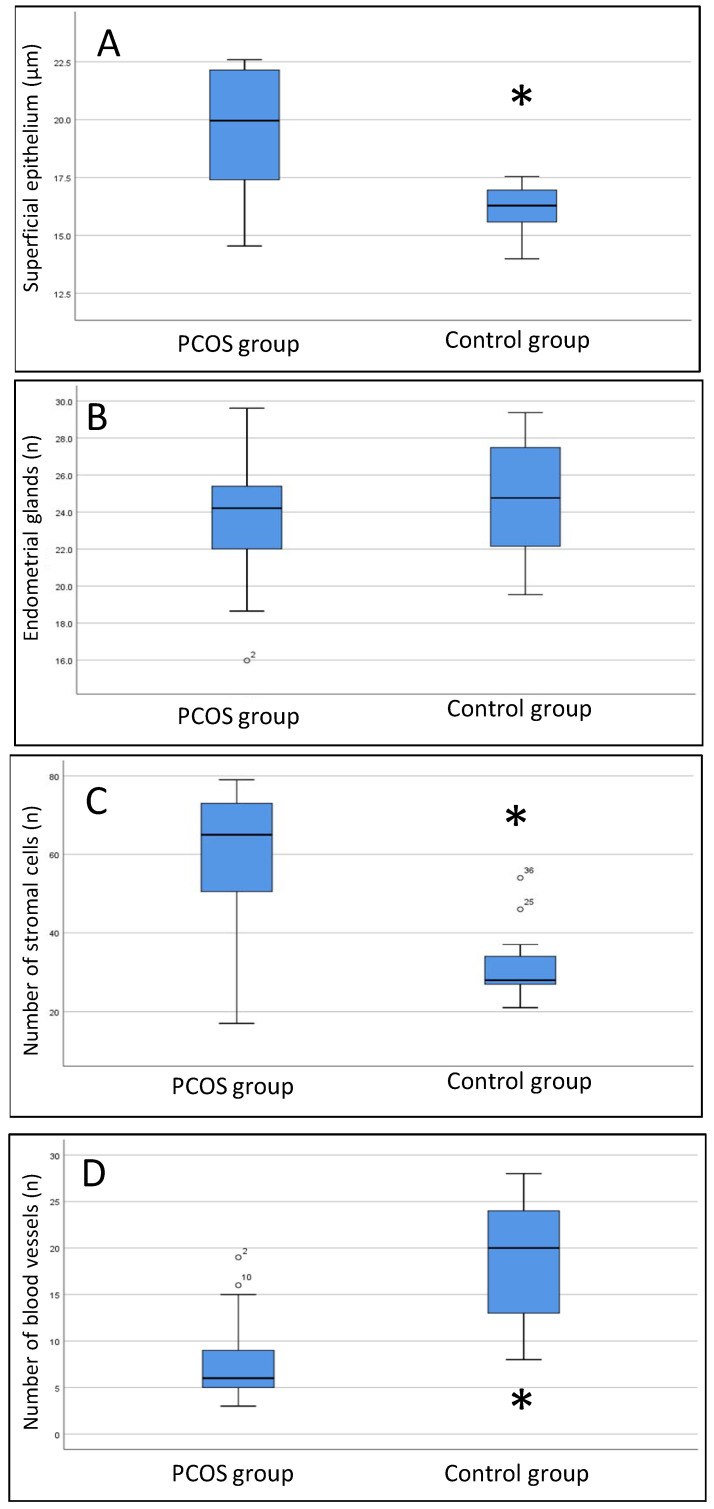
Box plot results of histomorphometric data. (**A**) thickness of the superficial epithelium; (**B**) number of endometrial glands; (**C**) number of stromal cells; (**D**) number of blood vessels. The Mann–Whitney *U* test was used. * PCOS group compared to the control group, *p* < 0.01.

The histomorphometric data of both groups obtained on days 21 and 24 of the cycle under progesterone treatment are shown in Figure 5. We noted a thicker surface epithelium in women with PCOS than in women without PCOS (median 20.1 [IIIQ {25–75 quartile interval} 17.5–22.1] vs. 16.3 [IIIQ 15.2–16.9]. *p* < 0.001). The number of endometrial cells in the controls (29.5 [IIIQ 26.3–33.8] was lower than that in women with PCOS, regardless of progesterone treatment (65 [IIIQ 44–72.5], *p* < 0.001). Conversely, the number of blood vessels was greater in controls than in women with PCOS (18 [IIIQ 12.3–23.8] vs. 7 [IIIQ 5–10], *p* < 0.001).

### 3.6. Spearman’s Correlation

Table 4 presents the correlations between histomorphometric findings and clinical and hormone measurements. The thickness of the surface epithelium was positively correlated with the percentage of free testosterone (FT) (r = 0.431, *p* = 0.009) and bioavailable testosterone (BioavT) (r = 0.429, *p* = 0.008) and negatively correlated with FSH (r = 0.374, *p* = 0.025). The thickness of the glandular epithelium correlated negatively with BMI (r = −0.393, *p* = 0.018), and the number of stromal cells correlated positively with baseline insulin (r = 0.435, *p* = 0.009), FT percentage (r = 0.434, *p* = 0.008), and BioavT (r = 0.436, *p* = 0.008), and negatively with FSH (r = −0.406, *p* = 0.014). Finally, blood vessel count was negatively correlated with baseline insulin (r = −0.344, *p* = 0.043), FT percentage (r = −0.348, *p* = 0.038), and BioavT (r = −0.350, *p* = 0.036).

## 4. Discussion

PCOS is a complex endocrine metabolic disorder that affects women during their reproductive years and can affect several organs and tissues. In the endometrium, PCOS is associated with an increased risk of miscarriages and proliferative lesions, and it can ultimately lead to endometrial cancer [1,2,3,4,19,27]. In fact, obesity and metabolic factors, such as insulin resistance, influence the endometrium, which may respond inadequately to progesterone treatment. In addition, free testosterone may interfere with endometrial progesterone-induced changes, resulting in the proliferation of epithelial and stromal cells and a decrease in the number of blood vessels. These histological features may indicate inadequate mucosa for embryo implantation [3,6,7,8,9,10,11,12,13,14,15,16,17,18,19,27,28,29].

In this study, the endometrium of PCOS patients during the first phase (after progesterone treatment) presented signs of cell proliferation compared with that of the control group, thereby suggesting that progesterone transformed the endometrium of women with PCOS. This may affect the action of progesterone [12]. We assumed worse outcomes in women with PCOS than in controls even after progesterone administration due to the previous endometrial proliferation linked to oestrogen action and the effects of insulin and androgen on the endometrium of such patients [4,6,9]. Biopsies of the first phase are important for evaluating the pre-status of the proliferative endometrium before progesterone treatment [4,9].

Lopes et al. [9] used natural progesterone at a dose of 200 mg/day orally for 10 days and found that this treatment did not promote the involution of endometrial changes in the PCOS group during the secretory phase. Therefore, in our study, we used a higher dose of natural progesterone (400 mg/day) and changed the route (vaginal route) (PCOS Study A). We verified that this dosage for 10 days was also insufficient to promote secretory features in the endometrium of a woman with PCOS compared with a control. Hence, carbohydrate metabolism disorders and excessive androgen production may underlie such an inadequate response [9]. Similarly, the androgen profile (total and free testosterone) may influence the endometrial transformation and low number of vessels, which are important for the nutrition of the embryo after implantation [13,14,15,17,18,20,30,31,32,33,34,35,36]. One mechanism that may explain the number of abortions in PCOS patients is the low quality of the endometrium [11,12,13,14,15,16,17,18]. Therefore, recommendations for weight loss from the international evidence-based guidelines for the assessment and management of polycystic ovary syndrome may be important not only for the amelioration of PCOS features, but also for the improvement of endometrial quality in response to progesterone treatment.

Although few studies have evaluated the regulation and molecular mechanisms involved in endometrial decidualisation, much is yet to be learned about the metabolic influence in this process. Besides the shrinking number of blood vessels, several other factors might modulate the endometrial response to micronized progesterone, such as the chronic inflammatory process [6,8,9,11,12,13,31]. In addition, this may explain why obesity is considered a factor that influences fertility [31,32,33]. Obesity may be due to gut microbiome disturbances and other metabolic mechanisms that affect endometrial quality and hinder the interaction between the embryo and endometrium [11,12].

Imprisoned senescent cells may hinder the action of younger cells in endometrial regeneration, thereby increasing the risk of endometrial dysfunction and encouraging the emergence of neoplastic cells [29,30,31]. This phenomenon may lead to persistent endometrial proliferation and is related to the prolonged action of insulin and androgen on the endometrium [3,6,9,14,19,29,30,31]. Therefore, classic progesterone treatment may be sufficient to revese previous hormonal and metabolic effects on the endometrium without decreasing body mass index.

It is unclear whether women with PCOS have an endometrium that is inadequate, notwithstanding an anovulatory state with reduced progesterone levels [11,12]. A few studies have indicated that the endometrium would no longer be propitious for implantation in PCOS following ovulation induction, given that term pregnancy rates remain low, even with letrozole administration, with its potentially lesser impact on the endometrium [34,35,36]. In addition, the uterine vascularity assessed by ultrasound at all sites was inadequate in the PCOS endometrium before and after clomiphene treatment [37], which may explain the low number of blood vessels in the PCOS endometrium under progesterone treatment. Furthermore, another study showed that differences in gene expression provided evidence of progesterone resistance in the midsecretory PCOS endometrium, independent of clomiphene citrate, and corresponding to the observed phenotypes of hyperplasia, cancer, and poor reproductive outcomes in women with PCOS [38]. However, the concern of that study was the low number of control patients (n = 3), which made it difficult to match the age and BMI of PCOS patients [38]. Nonetheless, our data suggest the need to better evaluate endometrial transformation using progesterone in women with PCOS and hyperandrogenemia. Other factors may also need to be corrected to increase the success rates of assisted reproductive programs [11,12,31,32,33,34,35], but mainly to improve the endometrial response to progesterone.

One limitation of our study was that we could not assess other factors that may influence endometrial transformation after progesterone treatment. Thus, a direct causality link between the cause-and-effect conclusions cannot be drawn. Consequently, studies on cell culture and animal models are necessary. In contrast, the strength of our study lies in the fact that a hormone disorder (hyperandrogenism) and a metabolic disorder (hyperinsulinemia) may interfere with adequate endometrial transformation caused by classic doses of natural progesterone.

Finally, in the endometrium of women with PCOS, treatment with natural progesterone (400 mg/day via the vaginal route for 10 days) was not sufficient for adequate endometrial transformation, which is a concern for the implantation process. Our results suggest that hormonal (hyperandrogenism) and metabolic (hyperinsulinemia) factors may interfere with the action of progesterone. Further studies are required to evaluate the molecular mechanisms involved in this process.

## 5. Conclusions

Our results suggest that histomorphometric abnormalities of the endometrium persist and are linked to androgen and insulin levels despite progesterone supplementation in PCOS.

## Figures and Tables

**Table 1:** Group-wise clinical and sociodemographic features.

Variables	PCOS (n = 28)	Control (n = 18)	* *p* Value
Age (Mean ± SD) *	27.41 ± 5.73	30.11 ± 5.33	0.118
Age at menarche (Mean ± SD) *	12.89 ± 1.87	12.50 ± 2.94	0.581
Marital status, n (%) **			
Married	21 (75)	9 (50)	
Single	6 (21.43)	7 (38.89)	
Divorced	1 (3.57)	2 (11.11)	0.078
Ethnicity, n (%) **			
White	12 (42.86)	8 (55.56)	
Afrodescent	16 (57.14)	10 (44.44)	0.577
Schooling, n (%) **			
Complete primary school	6 (21.43)	7 (38.89)	
Complete secondary school	15 (53.57)	6 (33.33)	
Complete College/University	7 (25)	5 (27.78)	0.319
Religion, n (%) **			
Catholic	9 (32.14)	8 (44.45)	
Evangelical	8 (28.57)	6 (33.33)	
Others	11 (39.29)	4 (22.22)	0.481
Family income **			
<2 minimum wages	18 (64.29)	10 (55.56)	
>2 minimum wages	10 (35.71)	8 (44.44)	0.387
Number of yearly menstrual cycles *	3.18 ± 2.21	11.61 ± 0.61	<0.001
Duration of flow (days) *	6.54 ± 4.36	5.44 ± 1.89	0.06
Number of pregnancies *	0.21 ± 0.42	0.72 ± 0.57	0.001
Number of childbirths *	0.21 ± 0.42	0.44 ± 0.61	0.176

SD = standard deviation. The *p* value was based on the Fisher exact test (qualitative) ** and the Mann–Whitney *U* test (quantitative) *.

**Table 2:** Group-wise anthropometric features and data on blood pressure and lipid and glucose metabolism.

Groups	PCOS (n = 28)		Controls (n = 18)		*p* Value
	Mean (±SD)	Median (P25–P75)	Mean (±SD)	Median (P25–P75)	
Anthropometric data					
BMI (kg/m^2^)	30.4 (±5.5)	30.3 (26–35.5)	30.5 (±5.2)	31.6 (28.3–33.6)	0.948
Abdominal circumference (cm)	103.9 (±14.8)	104 (94.5–114)	94.6 (±18.5)	95.5 (80.3–110.8)	0.065
Glucose metabolism					
Glycated hemoglobin (%)	5.7 (±0.3)	5.6 (5.1–5.8)	5.1 (±0.5)	5.2 (4.9–5.4)	0.001
GTT0 (mg/mL)	92 (±16.6)	87 (84.5–95.5)	88 (±8.1)	88.5 (81.3–93.5)	0.769
GTT30 * (mg/mL)	135 (±23.1)	138 (120.5–144.8)	110.1 (±20.3)	101 (94.5–127)	0.004
GTT60 * (mg/mL)	132 (±37.7)	131 (107–148.3)	104 (±19.2)	105 (93.3–117.5)	0.003
GTT90 * (mg/mL)	129 (±34.5)	131.5 (102.3–143)	102 (±23.1)	111 (81–120.5)	0.003
GTT120 * (mg/mL)	121.7 (±36.9)	119 (101.8–134.5)	105.1 (±20.1)	107 (90.5–120)	0.069
INS0 (µUI/mL)	41.1 (±41.4)	32.4 (14.8–42.9)	15.4 (±5.3)	15.5 (12–17)	0.002
INS30 * (µUI/mL)	172.9 (±90.1)	176 (109.8–211.3)	121.5 (±69.7)	115.5 (60.5–156)	0.06
INS60 * (µUI/mL)	186.6 (±97.6)	180.5 (119–245.8)	90.5 (±53.5)	83.5 (50.5–117.3)	<0.001
INS90 * (µUI/mL)	205.8 (±132.4)	178.5 (100.9–242.3)	107.6 (±52.9)	100 (67.8–147)	0.007
INS120 * (µUI/mL)	185.9 (±133.7)	155.3 (95.6–243.5)	101.9 (±55.3)	94.5 (59.3–120.5)	0.029
HOMA–IR	9.7 (±10.5)	6.5 (3.2–10.5)	3.4 (±1.3)	3.4 (2.5–3.7)	0.003
Matsuda Index	1.72 (±1.1)	1.3 (0.9–2.3)	3.41 (±1.18)	2.7 (2.2–4.2)	<0.001
Lipid metabolism					
Total cholesterol (mg/mL)	181.8 (±27.8)	172 (163.8–192)	185.4 (±28.9)	185 (175–195.5)	0.289
HDL-cholesterol (mg/mL)	39 (±13.6)	37 (26–48)	56.1 (±13.4)	57 (47–60)	0.001
LDL-cholesterol (mg/mL)	114.6 (±26.5)	107 (98.8–125.3)	112.9 (±33.1)	111 (92.5–131.3)	0.846
Triglycerides (mg/mL)	142.5 (±70.1)	130 (98–168.3)	93.6 (±43.1)	83. (61.8–117.3)	0.006

SD = standard deviation. The *p* value was based on the Mann–Whitney *U* test; oral glucose tolerance test (GTT) points: baseline, 30 min, 60 min, 90 min, and 120 min. Ins = insulin levels; insulin tolerance test (ITT) points: basal (INS0), 30 min, 60 min, 90 min, and 120 min. BMI, body mass index; BP, blood pressure; min, minutes; * values after glucose overload with 75 g of glucose.

**Table 3:** Ferriman–Gallwey index and laboratory data.

	PCOS (n = 28)		Control (n = 18)		* *p* Value
Groups	Mean (±SD)	Median (P25–P75)	Mean (±SD)	Median (P25–P75)	
F/G index (total score)	14 (±6.6)	11.5 (8–18)	5.1 (±0.3)	5 (4–5)	<0.001
Free testosterone e fraction (ng/dL)	1.3 (±0.6)	1.1 (0.8–1.7)	0.4 (±0.2)	0.3 (0.2–0.5)	<0.001
Percentage (%)	2.0 (±0.5)	2.0 (1.6–2.4)	1.8 (±2.3)	1.3 (1.0–1.7)	<0.001
BioavT (ng/dL) ^1^	29.3 (±14.6)	24.9 (18.6–39)	6.4 (±5.3)	6.4 (5.4–12.3)	<0.001
Percentage (%)	47.8 (±11.8)	47.8 (38.3–56.6)	29.3 (±10.8)	28.9 (23.7–37.7)	<0.001
SHBG (nmol/L)	30.3 (±14.7)	27 (18.8–39)	71.6 (±45.1)	58.5 (39.5–76.3)	<0.001
Total TESTO (ng/dL)	62.1 (±29.8)	57.5 (40.8–72.8)	28.1 (±8.1)	26.5 (22–32.8)	<0.001
DHEAS (µg/L)	1910 (±1157.2)	1713 (856–2528.8)	1475 (±557.8)	1477 (1385.8–1572.8)	0.351
A4 (ng/mL)	1.8 (±0.7)	1.8 (1.4–2.1)	1 (±0.4)	0.9 (0.8–1.2)	<0.001
Estradiol(pmol/L)	70.1 (±42.5)	65 (40.8–80.4)	57.6 (±28.3)	56.5 (35.5–77)	0.417
FSH (mUI/mL)	4.7 (±1.7)	4.5 (3.6–6.1)	6.2 (±1.4)	6.4 (5.9–7.1)	0.003
LH(mUI/mL)	9.9 (±4.6)	9.9 (5.6–12.9)	5.8 (±2.6)	5.4 (4.2–6.6)	0.001
PRL (µg/DL)	15.3 (±5.7)	14.5 (11–19.8)	14.9 (±4.9)	14 (11–18.2)	0.693

* The *p* value was based on the Mann–Whitney *U* test; F/G index = Ferriman–Gallwey index. ^1^ Bioavailable testosterone (ng/dL). DHEAS = dehydroepiandrosterone sulfate; A4 = androstenedione; PRL = prolactin; TESTO = testosterone; SHBG = S = sex hormone binding globulin.

**Table 4:** Correlation between the histomorphometric findings of hirsutism and the clinical and hormone measurements according to the histomorphometric analysis.

Variables	Surface Epithelium	Glandular Epithelium	Stromal Cell Number	Blood Vessels
Oral glucose tolerance test				
Correlation coefficient	−0.082	−0.189	−0.010	0.001
*p* value	0.633	0.269	0.953	0.994
Fasting insulin				
Correlation coefficient	0.229	0.028	0.435 **	−0.344 *
*p* value	0.185	0.875	0.009	0.043
BMI				
Correlation coefficient	−0.217	−0.393 *	−0.225	0.150
Abdominal circumference				
Correlation coefficient	−0.070	−0.139	0.048	−0.086
*p* value	0.691	0.425	0.785	0.624
Abdominal circumference/height ratio				
Correlation coefficient	−0.078	−0.214	0.062	−0.108
% Free testosterone				
Correlation coefficient	0.431 **	−0.139	0.434 **	−0.348 *
*p* value	0.009	0.419	0.008	0.038
Bioavailable testosterone				
Correlation coefficient	0.429 **	−0.140	0.436 **	−0.350 *
*p* value	0.008	0.414	0.008	0.036
FSH				
Correlation coefficient	−0.374 *	−0.131	−0.406 *	0.289
*p* value	0.025	0.447	0.014	0.087
LH				
Correlation coefficient	0.284	−0.162	0.274	−0.122
*p* value	0.093	0.345	0.106	0.478
SDHEA				
Correlation coefficient	0.026	0.178	0.274	−0.085
*p* value	0.882	0.299	0.106	0.622

Spearman’s correlation was applied for continuous clinical and laboratory variables with histological data. * negative correlation, ** positive correlation.

## Data Availability

Data available in a publicly accessible repository that does not issue DOIs.
